# Identification of a Cancer Stem Cells Signature of Head and Neck Squamous Cell Carcinoma

**DOI:** 10.3389/fgene.2022.814777

**Published:** 2022-05-11

**Authors:** Zeng-Hong Wu, Cheng Li, You-Jing Zhang, Wen Zhou

**Affiliations:** ^1^ Department of Otorhinolaryngology, Union Hospital, Tongji Medical College, Huazhong University of Science and Technology, Wuhan, China; ^2^ Department of Otolaryngology Head and Neck Surgery, The Central Hospital of Wuhan, Tongji Medical College Huazhong University of Science and Technology, Wuhan, China; ^3^ Department of Epidemiology, State Key Laboratory of Cardiovascular Disease, Fuwai Hospital, National Center for Cardiovascular Diseases, Chinese Academy of Medical Sciences and Peking Union Medical College, Beijing, China

**Keywords:** cancer stem cells, HNSCC, data mining, prognosis, gene expression

## Abstract

**Background:** Head and neck squamous cell carcinoma (HNSCC) ranks as the sixth most widespread and deadly cancer. In recent times, it has been determined that undifferentiated cell populations with stem cell-like properties in HNSCC are major factors influencing recurrence and progression.

**Method:** In this study, we determine key genes related to stemness by merging WGCNA with HNSCC mRNAsi based on the online database.

**Results:** We first download the mRNA expression-based stemness index (mRNAsi) data and contrast the expression levels of mRNAsi in cancers and control samples; we found significantly elevated mRNAsi expressions in HNSCC tissues (*p* = 0.002). Moreover, the brown module showed a relatively high negative correlation with mRNAsi (cor = -0.8). Thus, we selected the brown module as the interesting module and used it for following analysis. We screened 20 key genes (PDGFRB, PLPP4, CALU, ADAMTS14, COL5A3, KCNE4, LOXL1, CLEC11A, PODN,BGN, AEBP1, COL1A2, LAMA4, LOXL2, LRRC15, THY1, SPON2, COL1A1, NID2, and AC134312.5) including and as to decide the neighbor genes biological interaction network of these 20 stemness-related genes in HNSCC. The top 10 frequent alterations were PIK3CA, FGF3, FGF19, FGF4, DVL3, P3H2, GNB4, COL22A1, COL14A1, and PLOD2.

**Conclusion:** This study showed the critical role of stemness-related genes in HNSCC. However, more related studies are needed to confirm these results.

## Introduction

Head and neck squamous cell carcinoma (HNSCC), occurring in the oral cavity, lip, paranasal sinuses, larynx, and the nasopharynx, is a malignant tumor of the head and neck (Fang et al., 2018). It is the sixth most common type of malignancy, with more than 887,000 cases and 450,000 deaths every year (Ahmed et al., 2020). The known hazard factors for HNSCC include cigarette smoking, human papilloma virus (HPV) infection, and alcohol consumption (Magnes et al., 2017). Regrettably, there are few clinical manifestations in the early stages of cancer, leading to the majority of HNSCC patients being diagnosed at a later stage. As a result, the 5-years survival rate is less than 50%, and the survival rate for patients with local recurrence and metastasis is even lower, at 35% (Chin et al., 2005). Once advanced, treatment may affect organ function and impair speech and swallowing, resulting in a loss of quality of life (Coskun et al., 2015; Bressan et al., 2016).

In recent times, it has been determined that undifferentiated cell populations with stem cell-like properties in HNSCC are major factors influencing recurrence and progression. Cancer stem cells (CSCs) are a few highly tumorigenic cancer cells that are self-renewing and capable of differentiating into cells that make up most tumors (Patel et al., 2014). The resistance of current treatments, such as radiation therapy and chemotherapy, is attributed to the ability of the CSC subgroup to coordinate recurrence and promote metastasis, which is important for treatment (Davis et al., 2010). A recent study found that overexpression of retinoid X receptor-α (RXRα) can extend the CSC-like properties of HNSCC cells, while knockdown of RXRα can inhibit stem cells, whereas Wnt signaling pathways are induced by cisplatin in CSCs (Jiang et al., 2018). Similarly, the study also reported that *MTA3* can inhibit tongue squamous cell carcinoma (TSCC) CSC characteristics and tumor growth by down-regulating key regulators of CSCs SOX210 plasticity (Yao et al., 2019). In addition, the study also found that *YAP1/SOX2* transcriptional stress triggers activation of reprogrammable HNSCC to obtain stems, while triple *SOX2*, *CD44v9,* and *YAP1* may be useful for selecting high-risk CSCs with normal function (Omori et al., 2019). Overexpression of *PLOD2* enhanced CSC-like properties of Hep-2, activated Wnt signaling pathway, and activated laryngeal squamous cell carcinoma (LSCC) resistance *in vitro* and *in vivo* (Sheng et al., 2019). In this era of big data, bioinformatics analysis and microarray technology have been extensively used to simultaneously identify thousands of genes data at the genomic level. A study was carried out to quantify stemness based on a variety of platform analyses integrated methylation and transcriptome and transcription factor binding sites. mRNA expression-based stemness index (mRNAsi) from several tumors were collected, so mRNAsi data were downloaded for in-depth analysis (Pan et al., 2019). Higher mRNAsi scores are related to greater tumor dedifferentiation and active biological processes in CSCs, as reflected by histopathological grades. Weighted Gene Co-Expression Network Analysis (WGCNA) identifies a co-expressed set of genes, a collection called a module that can be correlated with phenotypic data to explore potential marker genes (Langfelder and Horvath, 2008). In this study, we make use of RNA sequencing (RNA-seq) data from the cancer genome atlas (TCGA) and determine key genes linked to stemness by combining WGCNA with HNSCC mRNAsi. Our findings will be of great significance in expounding the pathogenesis of CSC-related genes in HNSCC and in selecting useful biomarkers.

## Methods

### Gene Information and Bioinformatics Analysis

Information about gene expression (529 tissues) and comparative clinical data (378 cases) were downloaded from the TCGA-HNSCC cohort. The clinicopathological data were also collected. Data were examined with the R (version 3.5.3). We use Perl language for data matrix and data analyzing based on the *p* < 0.05. Missing data is processed using a table-by-list deletion technique, where if any single value is missing, the data excludes the entire sample from the analysis. mRNAsi is an index describing the level of similarity among stem cells and tumor cells and thus can be measured as a quantitative indication of CSCs. Logistic regression, Wilcoxon signed rank test, and Kruskal test were used to examine the relationship between clinical factors and mRNAsi.

### Identification of DEGs

The differentially expressed genes (DEGs) among HNSCC and non-cancerous samples was determined using the “edgeR” R package. To correct for the limitations of statistically significant gene discovery and false positives, we used the adjusted *p* values and the Benjamini and Hochberg false discovery rate. |log_2_FC| > 1 and *p* values < 0.05 were used as having statistical significance. The combat function of the “sva” R package was used to remove the batch effects.

### WGCNA and Module Preservation

WGCNA describes the connection among genes across the whole microarray sample, as well as the correlation between highly related genes or clusters of modules and outer conditions or sample traits, which make known the successive nature of the underlying co-expressed data and is set by cutoff parameters against data loss (Altermann and Klaenhammer, 2005). RNA sequence information was filtered to reduce outliers. Next, a weighted adjacency matrix was constructed and then transformed the adjacency matrix into a topological overlap matrix (TOM), which can assess the immediate connection of gene pairs and the intensity of a relationship with other genes in the dataset. The appropriate minimum gene module size for the gene tree was set to integrate close genes into a single module. Furthermore, a modular eigengene (ME), which can be representative of a modular gene expression profile, is determined as the first major component of the module of interest. In order to define the importance of each module, gene significance (GS) was used to measure the relationship among genes and sample traits. We chose mRNAsi and epigenetically regulated mRNAsi as clinical phenotypes. Typically, a marker gene is a highly connected central node that is at the heart of the network architecture. For every single gene, a module membership (MM) is defined by relating a given gene expression profile with the ME of a specific module. The cutoff for selecting hub genes in the module is characterized as cor. gene MM > 0.8 and cor. gene GS > 0.5.

### Enrichment Analyses

The Kyoto Encyclopedia of Genes and Genomes (KEGG) (Ashburner et al., 2000) is a tool for exploring high-expression gene functions and linking genomic data from extensive molecular datasets. Gene ontology (GO) (Szklarczyk et al., 2015) function analysis (biological processes (BP), cellular components (CC), and molecular functions (MF)) is a strong bioinformatics way to determine biological process and annotate genes. To explore the function of the determined DEGs, enrichment analyses were carried out based on the GO and KEGG pathway analysis by R language ggplot2 package to visualization figures.

### Protein-Protein Interaction Network Construction

STRING online database was used to predict the potential PPI network information (Gao et al., 2013). Analysis of the connection and function among DEG can give information on the mechanism of disease occurrence and development (PPI score >0.4). The number of adjacent nodes of individual gene was counted, and the genes were sorted on the basis of the number of adjacent nodes.

### Co-Expression Genes Analysis of Stemness-Related DEGs

cBioPortal (Visvader and Lindeman, 2008) is a free public asset that analyzes large-scale cancer genomics datasets. We applied c-BioPortal to explore changes in stem-associated DEG in TCGA-HNSCC samples and to provide a general view of the genetic changes for single test of stem-related DEG. The tab bio-interaction network of stem-associated DEGs and their co-expressed genes was examined and included adjacent genes with altered frequencies.

### Stemness Prognosis Related Genes in HNSCC

Kaplan-Meier plotter was applied to predict the prognostic significance of identified stemness-related genes from the key modules. We next explored the expression of stemness prognosis related genes across different cancer types based on the TIMER database and The Human Protein Atlas database (HPA) database. TargetScan predicts biological targets of miRNAs. Based on microRNA target prediction, free online tools from Diana-miRPath were used to assess interactions among miRNA previously determined using prediction tools and stemness prognosis related genes. We finally identified stemness-related lncRNA according to a correlation coefficient |*R* ^*2*^|>0.3 and *p* < 0.001.

## Results

### mRNAsi and Clinical Characteristics in HNSCC

**FIGURE 1:**
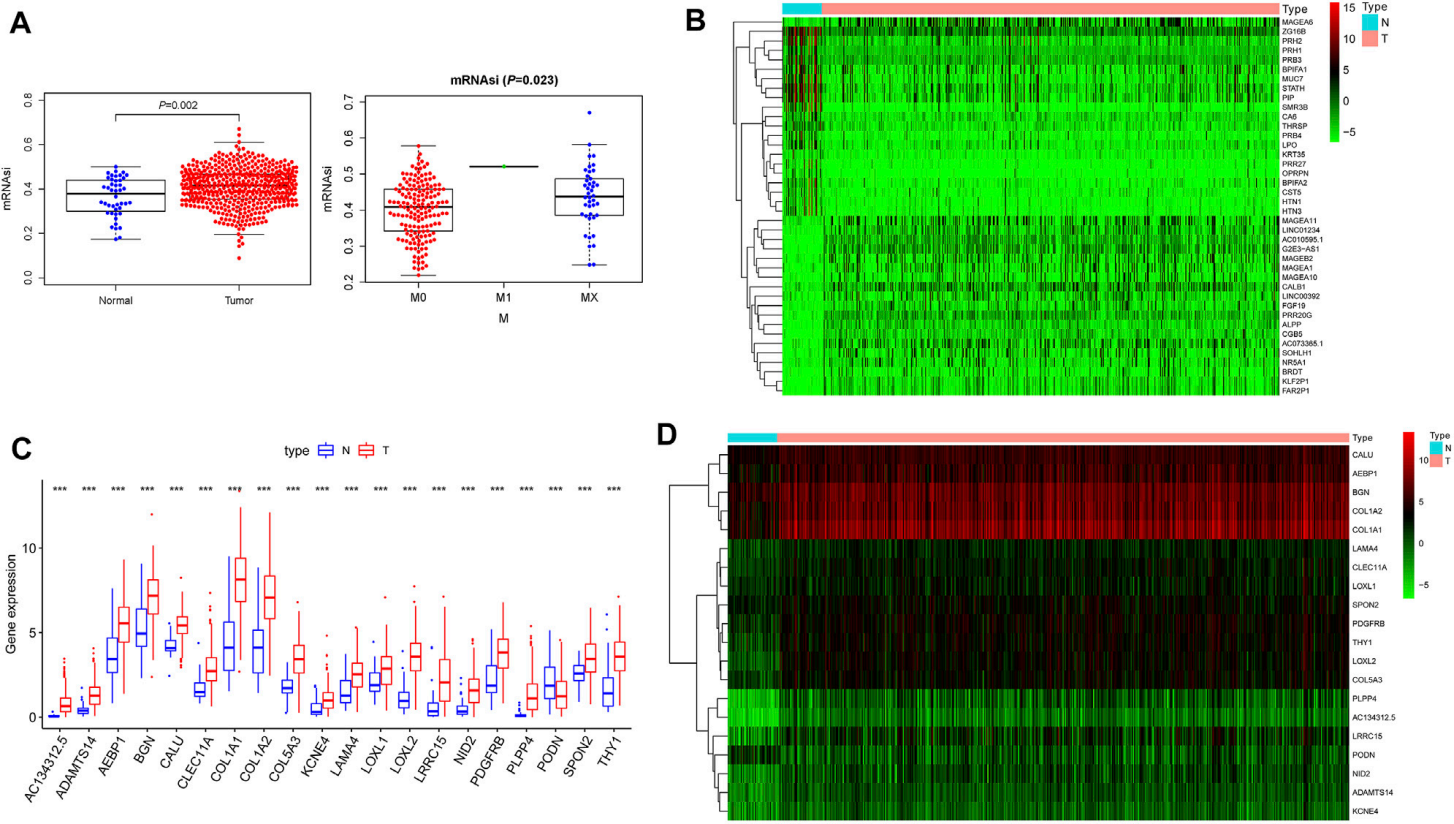
The characteristics of the mRNAsi. **(A)**. The connection of clinical factors and mRNAsi expressions and our results demonstrated that mRNAsi correlated significantly with the patient M-classification; **(B)**. The heatmap of the top 20 upregulated and downregulated differentially expressed genes of 4,732 DEGs between the HNSCC and normal samples; **(C)**. The difference expression transcriptional levels of these 20 key genes in cancers and normal samples; we found all key genes to have significantly higher expressions in HNSCC tissues; **(D)**. The heatmap of 20 key genes in the brown module.

We first downloaded the mRNAsi data from the statistics obtained by Pan *et al* and contrasted the transcriptional degrees of mRNAsi in tumors and control samples; we found significantly higher mRNAsi levels in HNSCC tissues (*p* = 0.002). We then explored the relationship of clinical elements and mRNAsi expressions and our results demonstrated that mRNAsi linked to the patient M-classification (*p* = 0.023) Figure 1A. The outcomes indicated that the expression of mRNAsi had significant difference and may play a vital part in regulating HNSCC. As normal mRNAsi is remarkably dissimilar from the tumor, we carried out data cleansing to find differential genes. Data normalization, filtering, and difference analysis were carried out to contrast HNSCC and normal samples. Thus, 4,732 DEGs were identified, of which 1,160 were downregulated and 3,572 were upregulated Supplementary Table S1. In addition, the heatmap of the top 20 upregulated as well as downregulated differentially expressed of DEGs was shown in Figure 1B.

### WGCNA Construction and Module Analysis

**FIGURE 2:**
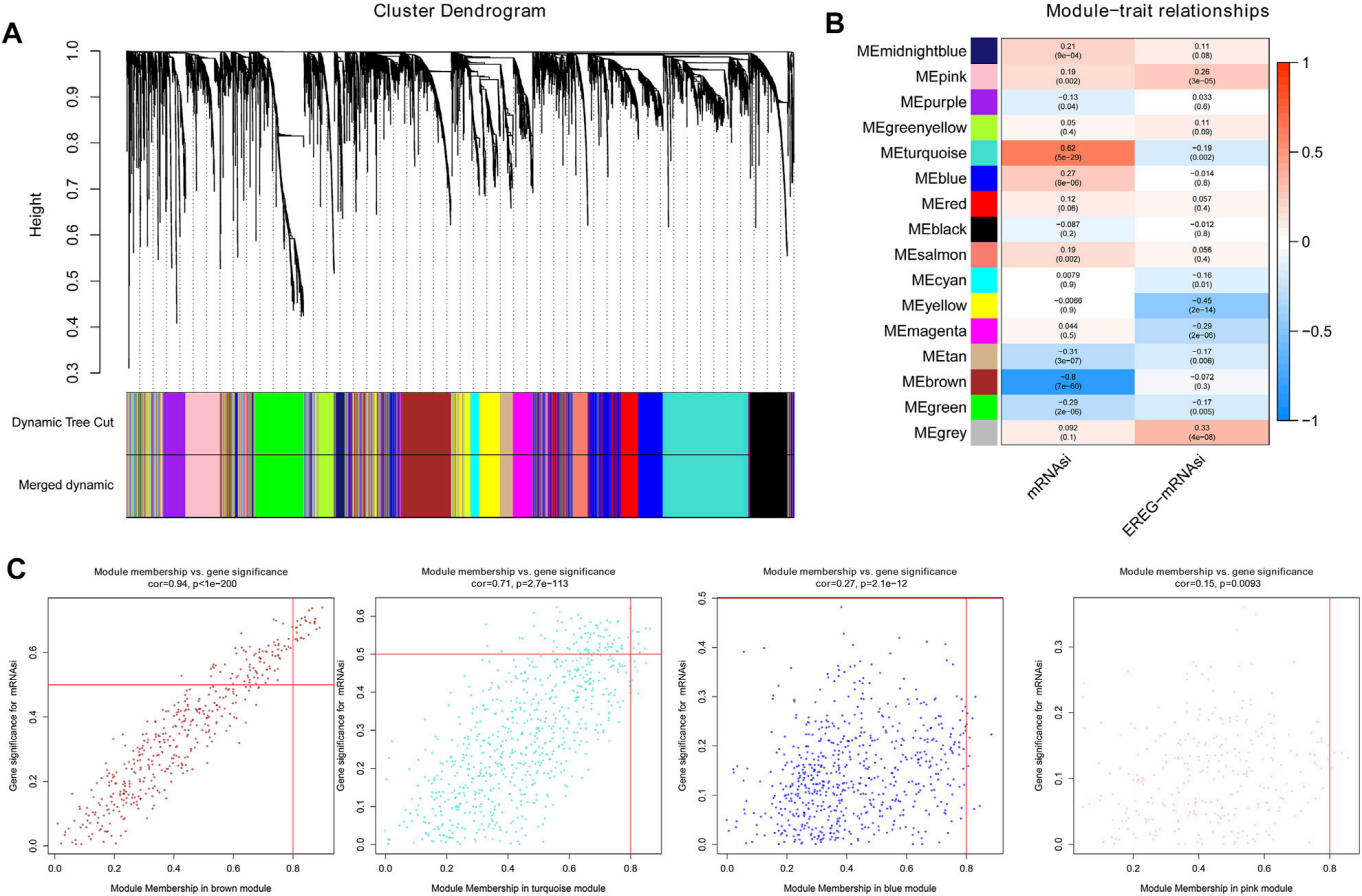
The results of the WGCNA analysis. **(A)**. By cluster analysis, DEGs with a variance of up to 25% were placed in one module, and we obtained 16 modules for the following analysis; **(B)**. The turquoise module was most remarkably relevant to mRNAsi, with a correlation near to 0.7. Moreover, the brown module showed a relatively high negative correlation with mRNAsi, with a correlation to -0.8; **(C)**. Scatter plot of module eigengenes in the blue, brown, pink, and turquoise modules.

Using cluster analysis, the DEGs with a maximum variance of one quarter were placed in one module, and we obtained 16 modules for the consequence analysis Figure 2A. The turquoise module was most correlated with mRNAsi, and the correlation coefficient was close to 0.7. In addition, the brown module shows a relatively high negative correlation with mRNAsi (cor = -0.8, Figure 2B). A scatter plot of module eigengenes in the blue, brown, pink, and turquoise modules is shown in Figure 2C. Thus, we select the brown module as the most interesting module and used it for the following analysis (Bai et al., 2020). We screened 20 key genes: *PDGFRB, PLPP4, CALU, ADAMTS14, COL5A3, KCNE4, LOXL1, CLEC11A, PODN, BGN, AEBP1, COL1A2, LAMA4, LOXL2, LRRC15, THY1, SPON2, COL1A1, NID2,* and *AC134312.5*.

### Functional Enrichment Analysis of Key Genes

**FIGURE 3:**
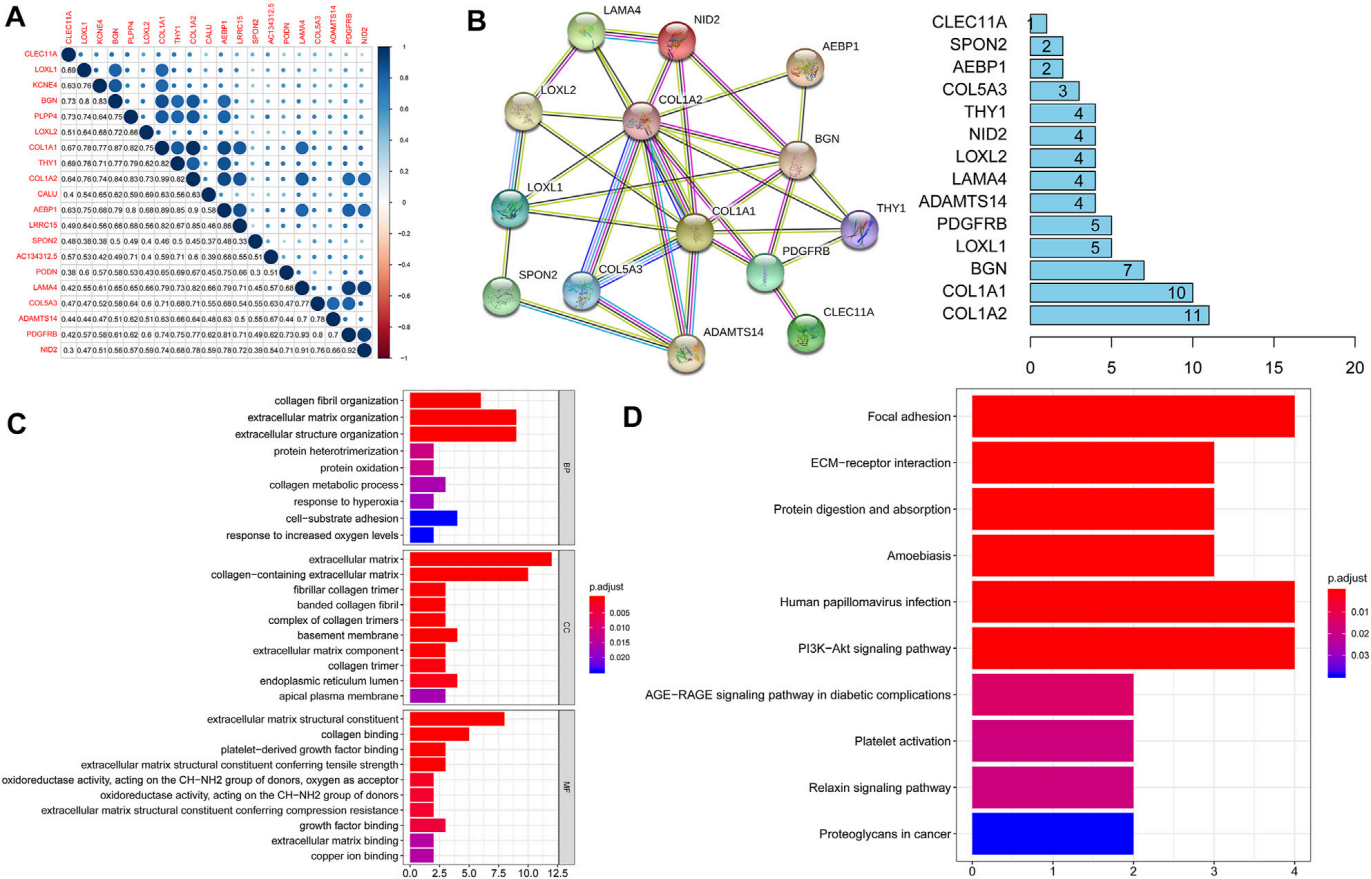
**(A)**. Function analysis of the DEGs. The co-expression of these 20 key genes showed the correlation with the lowest connection was between NID2 and CLEC11A (0.3), while that with the highest connection was between COL1A1 and COL1A2 (0.99); **(B)**. STRING database was used to construct the protein-protein interaction network and the number of adjacent nodes of each gene showed by histogram; **(C)**. GO analysis results of 20 key genes in the brown module; **(D)**. KEGG analysis results of 20 key genes in the brown module.

**FIGURE 4:**
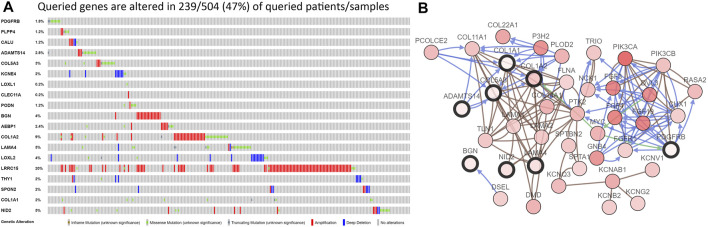
The results of the cBioPortal analysis. **(A)**. The 20 key genes were modified in 239 of 504 (47%) HNSCC patients and amplification is the most common modifications type in HNSCC; **(B)**. The neighbor genes’ biological interaction network of these 20 stemness-related genes in HNSCC.

We first analyzed the expression of these 20 key genes in cancers and normal samples and we found all key genes had significantly higher expressions in HNSCC Figure 1C; the heatmap of these 20 key genes is demonstrated in Figure 1D. We next analyzed the co-expression of these 20 key genes and we found the correlation with the lowest connection was between *NID2* and *CLEC11A* (0.3), while that with the highest interaction was among *COL1A1* and *COL1A2* (0.99) Figure 3A. We next constructed the PPI network and the count of adjacent nodes of single gene showed by histogram Figure 3B. Based on the histogram map, we found the most adjacent nodes gene was *COL1A1* and *COL1A2,* which indicated that these two genes may be key genes in the network. As to determine the neighbor genes’ biological interaction network of these 20 stemness-related genes in HNSCC, tab Network in cBioPortal and the 50 most as often altered neighbor genes were displayed and the top 10 frequent alterations were *PIK3CA, FGF3, FGF19, FGF4, DVL3, P3H2, GNB4, COL22A1, COL14A1, and PLOD2* (Figure 4B and Table 1). Meanwhile, these 20 key genes were modified in 239 of 504 (47%) HNSCC patients and amplification is the most usual modification type in HNSCC (Figure 4A).

**TABLE 1:** The top 10 type and frequency of mRNAsi related neighbor gene alterations in HNSCC (cBioPortal).

Gene symbol	Amplification	Homozygous	Mutation	Total
		Deletion		Alteration
PIK3CA	20.8	0	18.1	34.5
FGF3	24.8	0.2	0.2	25.2
FGF19	24.4	0.4	0.6	25.2
FGF4	24.6	0.4	0	25
DVL3	20.6	0	1.6	22.2
P3H2	21.4	0	0.6	21.6
GNB4	20.4	0	0.4	20.6
COL22A1	10.7	0	7.5	16.3
COL14A1	8.7	0	5.4	13.7
PLOD2	12.3	0	1.8	13.7

### The Functional Annotation of Brown Modules

To illuminate the functional similarity of the module genes, gene enrichment was performed based on the “clusterProfiler” R software package. GO analysis results indicated that changes in BP of 20 stemness-related genes were remarkably enriched in protein heterotrimerization, protein oxidation, collagen metabolic process, response to hyperoxia, cell-substrate adhesion, and response to increased oxygen levels. Changes in MF were mainly enriched in collagen binding, growth factor binding, extracellular matrix binding, and copper ion binding. Changes in CC of DEGs were mainly enriched in extracellular matrix, fibrillar collagen trimer, and apical plasma membrane Figure 3C. KEGG pathway analysis shows that the DEGs were mainly enriched in protein digestion, focal adhesion, ECM-receptor interaction and absorption, amoebiasis, Human papillomavirus infection, PI13-Akt signaling pathway, platelet activation, and proteoglycans in cancer Figure 3D. In addition, the DEGs were mainly enriched in ECM proteoglycans and the function of collagen based on the REACTOME pathway analysis Table 2.

**TABLE 2:** The function analysis of DEGs based on the REACTOME pathway analysis.

Term	Description	*p* Value (E)	Benjamini (E)
R-HSA-2243919	Crosslinking of collagen fibrils	3.8–7	1.4–5
R-HSA-3000178	ECM proteoglycans	2.9–6	5.5–5
R-HSA-3000171	Non-integrin membrane-ECM interactions	2.2–5	2.8–4
R-HSA-1650814	Collagen biosynthesis and modifying enzymes	1.0–4	9.9–4
R-HSA-1474244	Extracellular matrix organization	2.0–4	1.5–3
R-HSA-3000170	Syndecan interactions	6.5–4	4.1–3
R-HSA-2022090	Assembly of collagen fibrils and other multimeric structures	1.9–3	1.0–2
R-HSA-1442490	Collagen degradation	3.6–3	1.7–2
R-HSA-216083	Integrin cell surface interactions	6.5–3	2.7–2
R-HSA-75892	Platelet Adhesion to exposed collagen	2.1–2	7.4–2

### Analysis Stemness Prognosis Related Genes in HNSCC

**FIGURE 5:**
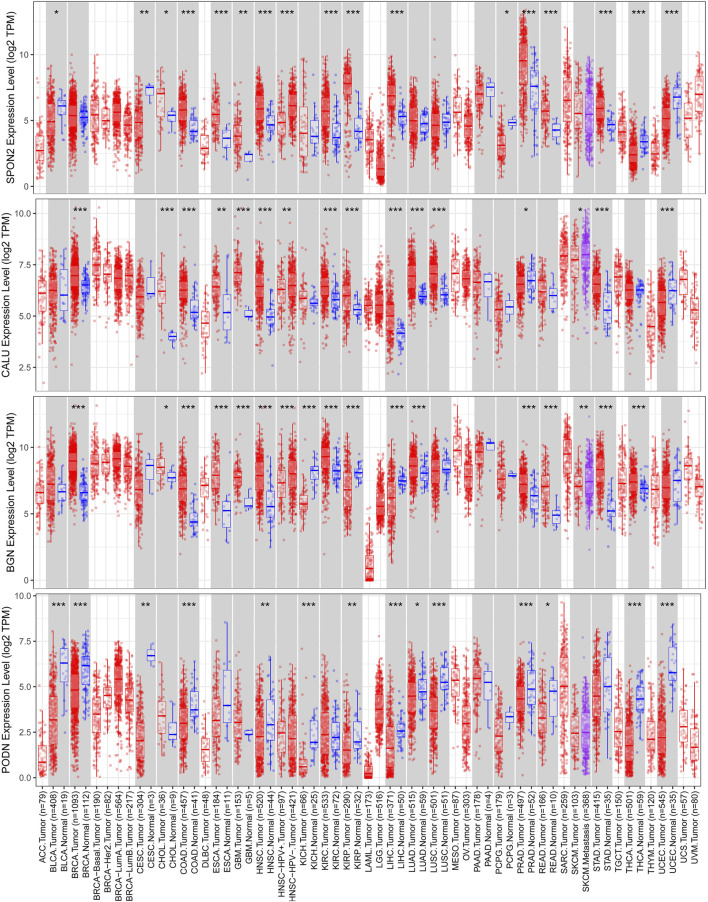
Multivariable Cox proportional hazard pattern showed that B cells, CD8^+^ T cells, and dendritic cells of immune infiltrates significant (*p* < 0.05) in HNSCC suggesting that these immune cells significantly stimulate the prognosis, it is significant for further exploration.

**FIGURE 6:**
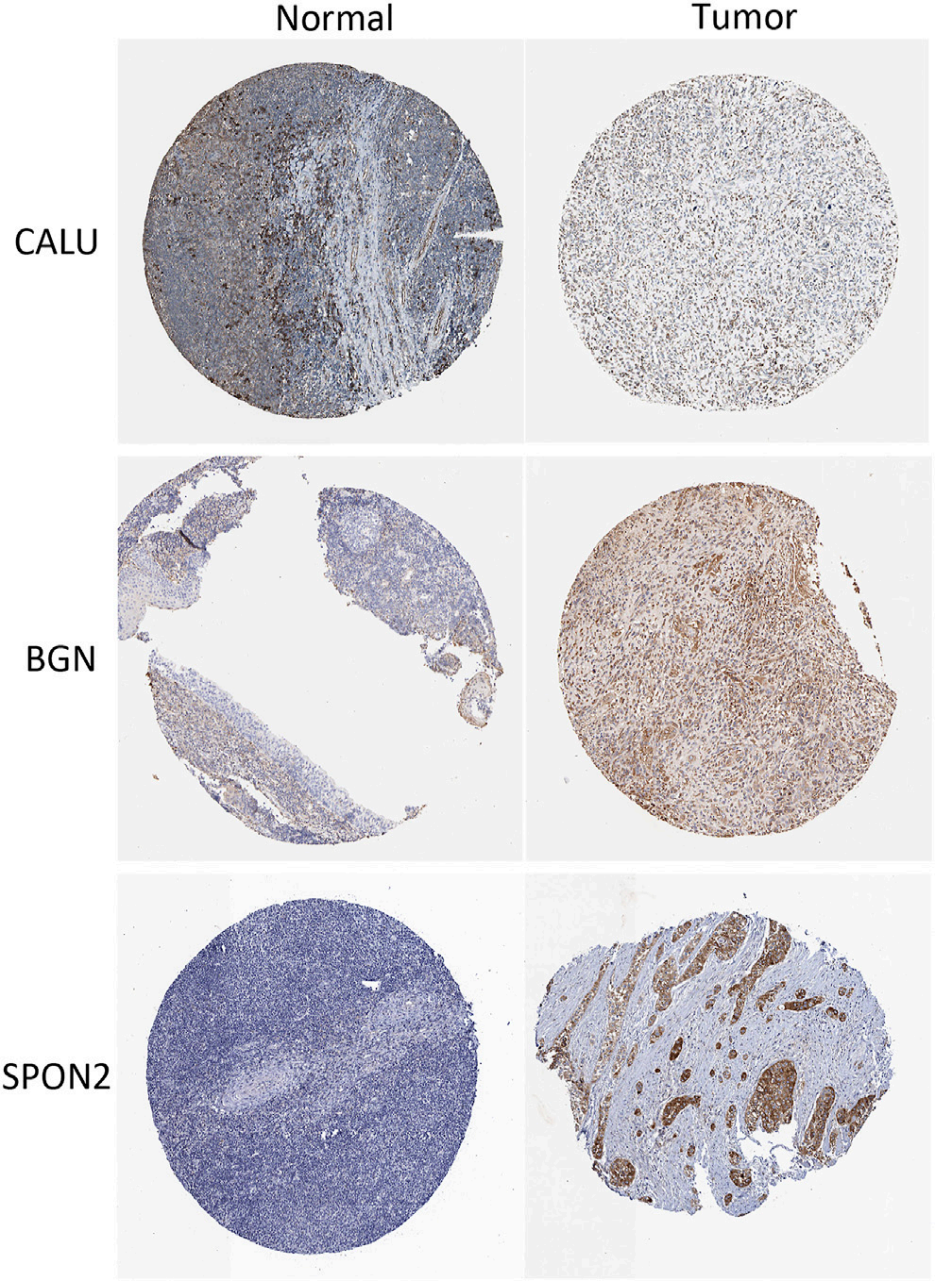
Immunohistochemistry results from the HPA database to illustrate that CALU, BGN, and SPON2 were significantly increased in tumor tissues.

**FIGURE 7:**
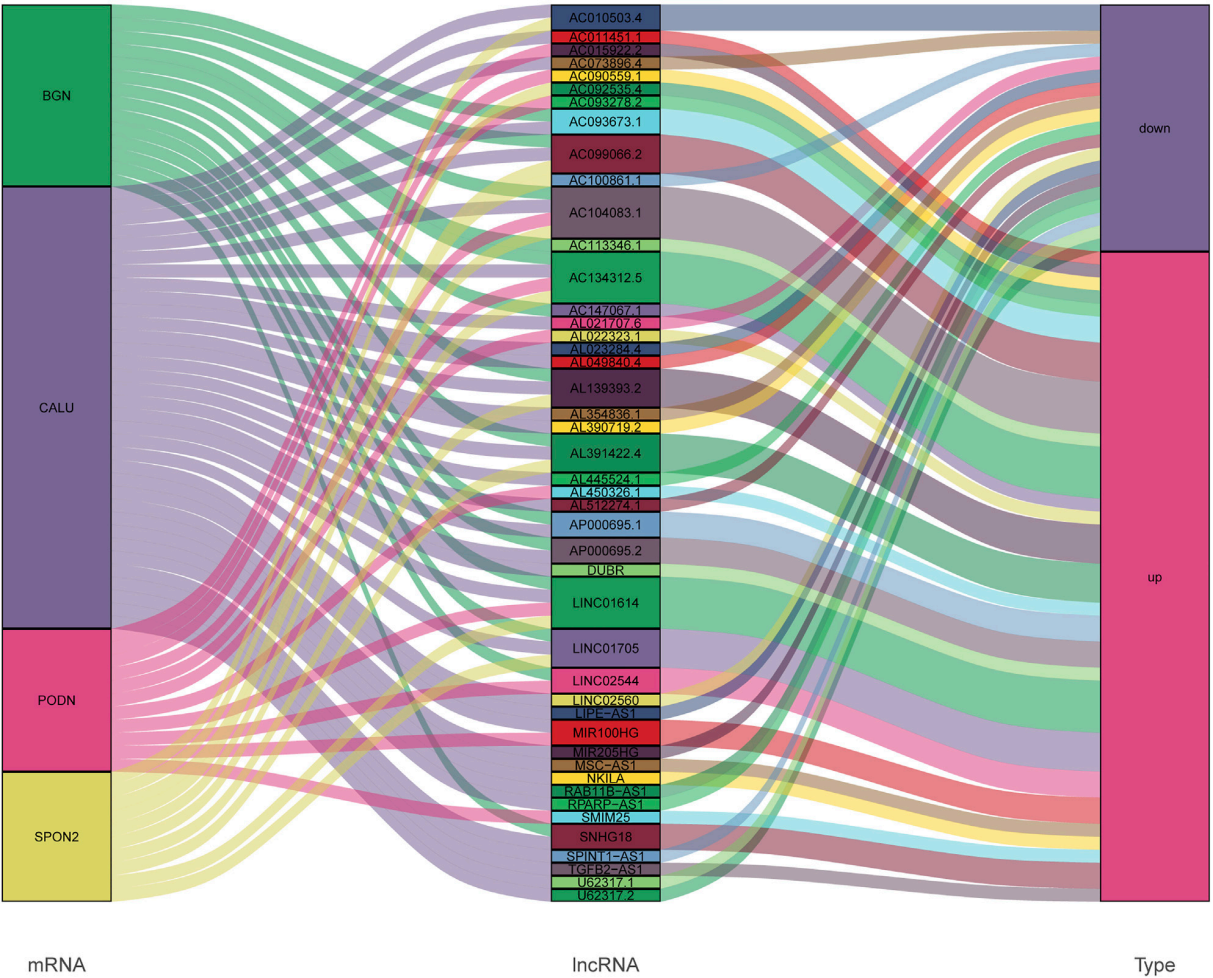
The relationship between the *CALU, BGN, POND, SPON2*, and lncRNAs.

**FIGURE 8:**
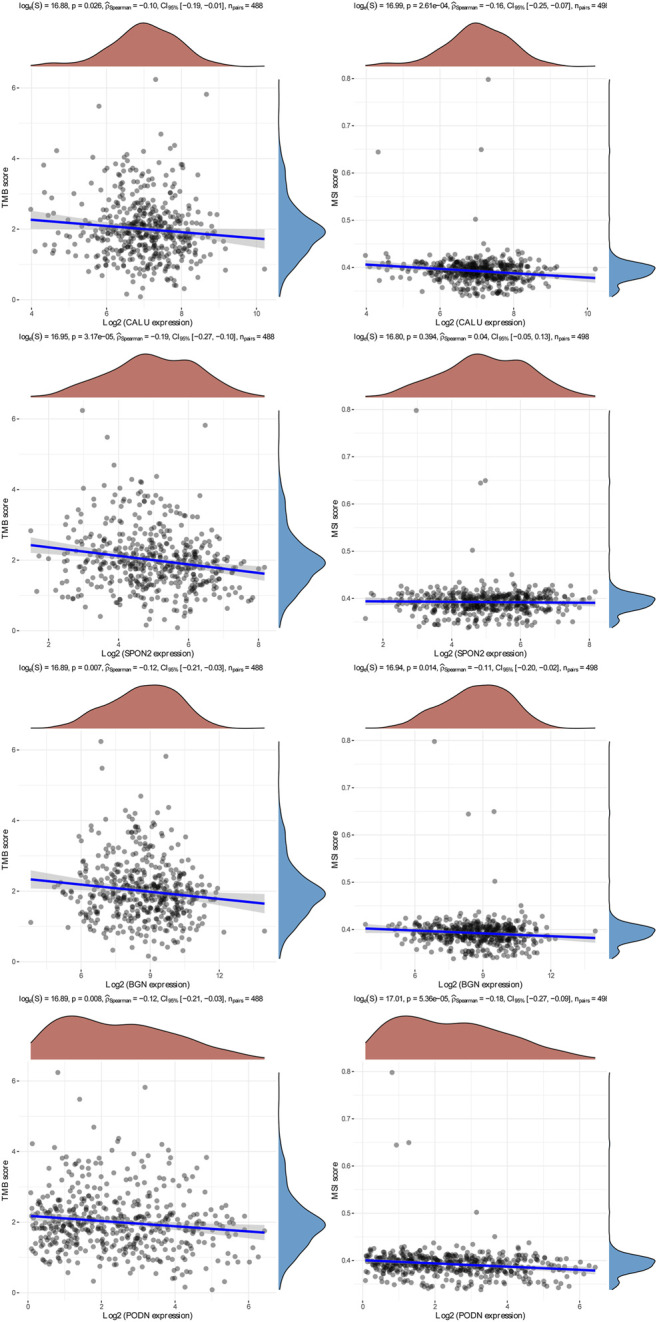
The connection among the *CALU, BGN, POND, SPON2,* TMB, and MSI.

We identified four stemness-related genes, *CALU, PODN, BGN,* and *SPON2,* related to overall survival in HNSCC from the brown module based on Kaplan-Meier plotter database Table 3. In order to further analyze the expression of these four genes, TIMER database was used to study the differential expression among tumor and adjacent normal tissues across all TCGA tumors Figure 5. Immunohistochemistry results from the HPA database was used to illustrate that *CALU, BGN,* and *SPON2* were significantly increased in tumor tissues (*POND* was missed) Figure 6. Prediction analysis using TargetScan tools determined the top 5 chosen miRNAs targeting each gene and these data help us understand how predicted miRNAs are linked to stemness-related HNSCC progress Table 4. There were 45 stemness-related lncRNAs determined according to the screening criteria. The lncRNA LIPE-AS1 had the largest correlation coefficient, and the target gene was *SPON2* (cor = 0.709; *p* = 1.57E-75; Supplementary Table S2). Meanwhile, the relationship between the four genes and lncRNAs were shown in Figure 7. Studies reported that tumor mutation burden (TMB) and microsatellite instability (MSI) plays an important role in tumor prognosis. Thus, we finally explored the connection among the 4 genes and TMB and MSI. The results shown that the TMB and MSI were significantly related to the stemness genes and need more in-depth research Figure 8.

**TABLE 3:** The overall survival in HNSCC from brown module based on Kaplan-Meier plotter database.

Genes	HR 95% CI	*p* Value
*PLPP4*	1.33 (1.00–1.76)	0.058
*PDGFRB*	0.75 (0.56–1.02)	0.067
*CALU*	1.46 (1.10–1.93)	0.0087
*ADAMTS14*	1.17 (0.89–1.55)	0.27
*COL5A3*	1.15 (0.86–1.53)	0.35
*KCNE4*	1.17 (0.88–1.56)	0.28
*LOXL1*	0.79 (0.60–1.03)	0.079
*CLEC11A*	1.27 (0.97–1.66)	0.082
*PODN*	0.74 (0.55–0.99)	0.045
*BGN*	1.33 (1.02–1.74)	0.035
*AEBP1*	1.24 (0.95–1.63)	0.11
*COL1A2*	0.86 (0.65–1.13)	0.28
*LAMA4*	0.80 (0.61–1.05)	0.11
*LOXL2*	1.32 (0.99–1.75)	0.56
*LRRC15*	1.15 (0.88–1.51	0.32
*THY1*	1.28 (0.98–1.67)	0.074
*SPON2*	1.36 (0.99–1.88)	0.045
*COL1A1*	1.20 (0.90–1.61	0.22
*NID2*	0.85 (0.65–1.11)	1.23
*AC134312.5*	NA	NA

**TABLE 4:** The Gene Ontology (GO) terms and Kyoto Encyclopedia of Genes and Genomes (KEGG) pathways enrichment among the four key genes.

Genes	Predicted miRNAs	Category		*p* Value
*CALU*	hsa-miR-1266–5p	GO terms	platelet degranulation (GO:0002576)	0.026
	hsa-miR-4518		sarcoplasmic reticulum (GO:0016529)	0.026
	hsa-miR-885–3p		platelet activation (GO:0030168)	0.031
	hsa-miR-4489			
	hsa-miR-596			
*PODN*	hsa-miR-6504–5p	KEGG pathway	Thyroid hormone synthesis (hsa04918)	5.025e-07
	hsa-miR-3064–5p		2-Oxocarboxylic acid metabolism (hsa01210)	0.0038
	hsa-miR-155–5p		Wnt signaling pathway (hsa04310)	0.0039
	hsa-miR-370–5p	GO terms	organelle (GO:0043226)	1.070e-09
	hsa-miR-1193		ion binding (GO:0043167)	2.650e-07
			cellular nitrogen compound metabolic process (GO:0034641)	1.415e-06
*BGN*	hsa-miR-2277–5p	GO terms	chondroitin sulfate catabolic process (GO:0030207)	0.0058
	hsa-miR-6885–5p		chondroitin sulfate metabolic process (GO:0030204)	0.0069
	hsa-miR-6777–5p		Golgi lumen (GO:0005796)	0.0071
	hsa-miR-5739		sulfur compound metabolic process (GO:0006790)	0.0198
	hsa-miR-6889–5p		extracellular matrix structural constituent (GO:0005201)	0.0091
*SPON2*	hsa-miR-1908–3p	GO terms	mast cell mediated immunity (GO:0002448)	0.0013
	hsa-miR-491–5p		induction of bacterial agglutination (GO:0043152)	0.0013
	hsa-miR-6746–5p		opsonization (GO:0008228)	0.0039
	hsa-miR-6081		lipopolysaccharide binding (GO:0001530)	0.0062
	hsa-miR-6827–5p		antigen binding (GO:0003823)	0.0077

## Discussion

In this study, based on the statistical data from Pan *et al.*, we conducted a thorough and full assessment of the prime genes associated with stemness by combing WGCNA with HNSCC mRNAsi based on the mRNAsi score, and explored their relationship with clinicopathological characteristics, function, and immune infiltration. CSCs have been suggested to participate in tumor progression, therapeutic resistance, and recurrence (Bai et al., 2020). So, therapeutic targeting of HNSCC stem cells is necessary. CSCs having a self-renewal ability as well as capacity to differentiate into various cell lineages contribute to progression, cancer initiation, and dissemination to distant organs (Tirino et al., 2013). CSC has an endogenous resistance mechanism to radiation and chemotherapy, which gives CSC a survival advantage over differentiated counterparts (Bao et al., 2006; Jeon et al., 2011). Clinically, typical therapies including surgery, radiation, and chemotherapy have been successfully applied to get rid of most cancer cells. But, as for the lack of useful targeted therapy against HNSCC-CSC, intrinsically resistant CSC regions cannot be retained, resulting in regeneration of cancer cells after treatment. Thus, the discovery of the potential novel stemness related genes is important. The acquisition of progenitor cell-like and stem-cell-like features and the loss of differentiated phenotypes are indicative of cancer progression, and the high level of HNSCC remains constant as the tumor progresses. Our results show that mRNAsi is significantly associated with the M classification of patients, suggesting that stem cell characteristics begin to derive from the initiation of metastasis.

The key genes were identified from the brown module derived from GS and MM; from the co-expression of this module we found the relationship with the lowest correlation was among *NID2* and *CLEC11A* (0.3), while that with the highest correlation was among *COL1A1* and *COL1A2* (0.99). Meanwhile, from the histogram map, we found the largest adjacent nodes gene was *COL1A1* and *COL1A2*, which indicated that these two genes may be the hub genes in the network. Collagen type I alpha 1 (*COL1A1*) belong to group I collagen which involves *COL1A1*/*COL1A2* (Chan et al., 2008). Studies have reported that *COL1A1* not only has high expression in gastric cancer but also plays key roles in cancer cell invasion and metastasis (Sun, 2016). A recent study found that *COL1A1* activation could suppress the apoptosis of cervical cancer cells (Liu et al., 2017). Ma *et al* (Ma et al., 2019) reported that *COL1A1* shows survival benefit and increases oncogenicity on hepatocellular carcinoma cells. In addition, *COL1A1* and *COL1A2* might forecast bad clinical results in gastric and colon cancer patient (Li et al., 2016; Zhu et al., 2020). Misawa *et al* (Misawa et al., 2011) found that CpG hypermethylation is a probable method of *COL1A2* gene inactivation, supporting the presumption that the *COL1A2* gene may play an indispensable role in the tumorigenesis of HNSCC. So, we recommended that *COL1A1* and *COL1A2* may serve as a possible prognostic biomarker for HNSCC prognosis and therapeutic targets, but more research is needed for further investigation.

To decide the neighbor genes’ biological interaction network of these 20 stemness-related genes in HNSCC, we identified the top 10 frequent alterations were *PIK3CA, FGF3, FGF19, FGF4, DVL3, P3H2, GNB4, COL22A1, COL14A1 and PLOD2.* Phosphoinositide 3-kinase (*PI3 K*) and serine/threonine kinase AKT pathway regulate cellular functions such as proliferation, cell survival, and differentiation (Engelman et al., 2006). *PIK3CA* mutation status was significantly related to median tumor size and significantly correlated to decreased disease-free survival and overall survival in cervical cancer (Chung et al., 2017). Kidacki *et al* (Kidacki et al., 2017) reported that *PIK3CA* mutations facilitate *MMP1*-driven invasion, which supplied a potential novel target for poor metastasis in HNSCC. A recent study found that the merge of a *PI3K/mTOR* inhibitor and palbociclib completely controlled tumor growth in mice (Zainal et al., 2019). We hypothesize that *PIK3CA* may contribute to the incidence of HNSCC and needs more related research.

HPV-related HNSCC has increased over the past 2 decades and now makes up most HNSCC cases (Koneva et al., 2018). Functions and pathways of 20 stemness-related genes were significantly enriched in Human papillomavirus infection, PI13-Akt signaling pathway. So, the stemness-related genes in the brown module may related to HNSCC-HPV *via* PI13-Akt signaling pathway and AGE-RAGE signaling pathway. Our study may contribute to future research into the mechanism of cancer incidence in HNSCC. We also explored the TMB and MSI correlation with stemness-related genes in HNSCC. Liao *et al* (Liao et al., 2013) reported that CSC populations were less sensitive to MHC class I-restricted alloantigen-specific CD8 (+). CTL lysis as contrasted to matched monolayer-derived cells needs more exploration**.** In total, 20 key genes were found to play key roles in HNSCC stem cell maintenance.

## Conclusion

Our study showed the dominant role of stemness-related genes in HNSCC. However, more related studies are needed to support these results and push forward the application of these key genes’ prognosis evaluation.

## Data Availability

The original contributions presented in the study are included in the article/Supplementary Material, further inquiries can be directed to the corresponding author.

## Supplementary Material

Supplementary Table S1 The expression data of DEGs in TCGA-HNSCC.

Supplementary Table S2 Stemness-related lncRNAs determined according to the screening criteria.

## Abbreviations

CSCs, Cancer stem cells; HNSCC, Head and neck squamous cell carcinoma; mRNAsi, mRNA expression-based stemness index; WGCNA, Weighted Gene Co-Expression Network Analysis.
